# Testing Factor Structure and Measurement Invariance Across Gender With Italian Geriatric Anxiety Scale

**DOI:** 10.3389/fpsyg.2018.01164

**Published:** 2018-07-05

**Authors:** Laura Picconi, Michela Balsamo, Rocco Palumbo, Beth Fairfield

**Affiliations:** ^1^Department of Psychological, Health & Territorial Sciences, University of Chieti, Chieti, Italy; ^2^Department of Neurology, Boston University, Boston, MA, United States; ^3^Centro Scienze dell'Invecchiamento e Medicina Traslazionale, University of Chieti, Chieti, Italy

**Keywords:** geriatric anxiety scale, late-life anxiety, factor structure, measurement invariance, gender differences

## Abstract

Late-life anxiety is an increasingly relevant psychiatric condition that often goes unnoticed and/or untreated compared to anxiety in younger populations. Consequently, assessing the presence and severity of clinical anxiety in older adults an important challenge for researchers and clinicians alike. The Geriatric Anxiety Scale is a 30-item geriatric-specific measure of anxiety severity, grouped in three subscales (Somatic, Affective, and Cognitive), with solid evidence for the reliability and validity of its scores in clinical and community samples. Translated into several languages, it has been proven to have strong psychometric properties. In Italy only one recent preliminarily investigative study has appeared on its psychometric properties. However, sample data was largely collected from one specific Italian region (Lombardy) alone. Here, our aim in testing the items of the GAS in a sample of 346 healthy subjects (50% females; 52% from Southern Italy), with mean age of 71.74 years, was 2-fold. First, we aimed to determine factor structure in a wider sample of Italian participants. Confirmatory factor analysis showed that the GAS fits an originally postulated three-factor structure reasonably well. Second, results support gender invariance, entirely supported at the factorial structure, and at the intercept level. Latent means can be meaningfully compared across gender groups. Whereas the means of F1 (Somatic) and F3 (Affective) for males were significantly different from those for females, the means for F2 (Cognitive) were not. More specifically, in light of the negative signs associated with these statistically significant values, the finding showed that F1 and F3 for males appeared to be less positive on average than females. Overall, the GAS displayed acceptable convergent validity with matching subscales highly correlated, and satisfactory internal discriminant validity with lower correlations between non-matching subscales. Implications for clinical practice and research are discussed.

## Introduction

Late-life anxiety is an increasingly relevant psychiatric condition and will become an increasing cause of health care utilization, contributing to elevated personal and societal costs, as numbers of older adults constantly increase in diverse countries across the developing world (Wolitzky-Taylor et al., 2010; Baxter et al., 2013). In Italy, for example, 7.3% of the older adults showed symptoms of chronic anxiety in 2013 (Istat, 2013). Additionally, due to a combination of declining fertility and increased life expectancy, the percentage of people older than 65 years will likely reach 33% of the total population by 2056 (Istat, 2011) and will further increase the percentage of chronic anxiety.

The detection of anxiety disorders in older adults, however, can be complicated by cognitive impairment, newly emergent changes in life circumstances, high age-related medical and psychiatric comorbidity, and a symptom presentation that is markedly different from younger age groups (Magni and DeLeo, 1984; Kogan et al., 2000; Cully et al., 2006; Seignourel et al., 2008; Balsamo et al., 2010; Wolitzky-Taylor et al., 2010; Therrien and Hunsley, 2012). For these reasons, late-life anxiety is more likely to go unnoticed and untreated compared to anxiety in younger populations and makes assessing the presence and severity of clinical anxiety in older adults an important challenge for researchers and clinicians alike. Nonetheless, relatively little is known about the assessment of anxiety in older adults (Ayers et al., 2007; Balsamo et al., 2018).

Among assessment methods adopted for anxiety assessment in both research and clinical practice, self-report measures are by far the most common (Alwahhabi, 2003; Dennis et al., 2007; Antony and Barlow, 2011). Self-report inventories are easy-to-use and time-saving tools for screening psychopathology, measuring the severity of illness, limit patient/participant burden, and for monitoring treatment outcome. Approximately 12 anxiety measures have been identified as frequently used for the assessment of anxiety in older adults (Therrien and Hunsley, 2012). Importantly, most of these measures were originally developed and validated in college samples and therefore lack specific norms and sufficient psychometric evidence for use with older adults. The remaining instruments are new measures created specifically for use with older adults, such as the Geriatric Anxiety Inventory (GAI; Pachana et al., 2007), the Adult Manifest Anxiety Scale-Elderly Version (Reynolds et al., 2003), and the Geriatric Anxiety Scale (GAS; Segal et al., 2010).

Among the age-specific instruments of anxiety, the GAS provided solid evidence for the reliability and validity of its scores in clinical and community samples of older adults in the US (Segal et al., 2010; Yochim et al., 2011, 2013). Already translated in many languages such as German, Persian and Chinese (Bolghan-Abadi et al., 2013; Gottschling et al., 2015; Lin et al., 2016), this questionnaire has been shown to have good psychometric properties among Italian community-dwelling older adults (Gatti et al., 2017). However, its factorial structure has not yet been well-investigated in a large geographically varied sample. Indeed, in the study by Gatti et al. (2017), sample data was largely collected from one specific Italian region (Lombardy) alone.

In light of its promising psychometric properties functioning, including its ability to capture several components of anxiety (somatic, affective, and cognitive symptoms), our study aims to investigate the factor structure of the Italian version of the GAS within the structural equation modeling (Confirmatory Factor Analysis) framework and, to assess internal consistency, convergent and discriminant validity with measures of anxiety, depression, and personality, in a large Italian sample of healthy community-dwelling older adults. The latter feature of this measure is most important, because it allows clinicians to easily assess whether a patient is experiencing primarily somatic symptoms versus affective or cognitive symptoms, and thus to conclude whether the symptoms are related to a physical health problem instead of an anxiety disorder (Yochim et al., 2011). Moreover, since theoretical and empirical studies have presented mixed results concerning gender differences in experiencing anxiety in older adults (Mueller et al., 2015), we conducted a multiple-group CFA to assess (configural, metric, and scalar) measurement invariance of the GAS and latent means differences across gender groups. Gender, in fact, is a variable which has been identified as a risk factor for anxiety (see, for example, De Beurs et al., 2000; McLean et al., 2011; Mueller et al., 2015). Specifically, women tend to report higher levels of anxiety than men. So, lower scores on GAS scales for males than for females were expected in this sample (Owens et al., 2000).

## Materials and methods

### Participants and procedure

Three hundred and forty-six community-dwelling older adults (50% females) from different regions in Italy, recruited from student family members, friends and volunteers, participated in the study. Mean age of the sample was 71.74 (*SD* = 6.78) years. Participants did not receive monetary reimbursement for participation. Exclusion criteria were the presence of current treatment for memory problems, head injuries resulting in hospitalization for more than 24 h and/or medical conditions that could potentially affect cognitive functioning (e.g., Alzheimer's disease, multiple sclerosis, and Parkinson's disease) and, thus, the ability to take the assessment. Moreover, all participants reported being in good mental and physical health.

Initially, 436 questionnaires were returned. Seventeen did not contain answers to all of the GAS items (showing 10% or more missing values). In addition, 73 univariate outliers were detected and removed from the initial dataset by using standard z-score (Tabachnick and Fidell, 2007). Considering levels of education, most participants (27.3%) had a High School diploma and 13.9% a university degree. Most participants came from Central (40.8%) and Southern Italy (52%). Participant characteristics are described in detail in Table 1.

**Table 1:** Demographic characteristics of participants (*n* = 346).

**Characteristics**	
**Age** (years); mean (SD)	71.74 (6.78)
**Sex; % (n)**
Men	50 (173)
Women	50 (173)
**Marital status; % (n)**	
Single	6.2 (20)
Married	68.6 (223)
Divorced	3.7 (12)
Separated	1.5 (5)
Live-in partner	0.6 (2)
Widowed	19.4 (63)
**Education; % (n)**	
Primary or lower	36.4 (123)
Upper secondary	22.5 (76)
High School diploma	27.3 (92)
University degree	13.9 (47)
**Italian geographic areas; % (n)**	
Northern Italy	2.4 (8)
Central Italy	40.8 (136)
Southern Italy	52 (173)
South-islands	4.8 (16)

*With the exception of gender, percentages were calculated on the number of subjects who answered questions: 325 for marital status, 338 for Education, 333 for Italian geographic areas*.

For the construct validation of the GAS dimensions, 345 participants from the larger sample also completed the Big-Five Questionnaire 2 (BFQ-2), 327 completed the Teate Depression Inventory (TDI) and 346 completed the Geriatric Anxiety Inventory (GAI). Each participant anonymously completed the questionnaire packet and gave informed consent prior to their inclusion in the study. The study was approved by the Psychological Science Departmental ethical committee at the University of Chieti. All participants provided written, informed consent, in accordance with the Ethical Standards of the Helsinki Declaration.

### Measures

#### Geriatric anxiety scale (GAS)

The GAS (Segal et al., 2010) is a 30-item self-report measure used to assess and quantify anxiety symptoms among older adults. Individuals are asked to indicate how often they have experienced each symptom during the immediately preceding week, including today. Respondents answer using a 4-point Likert scale ranging from 0 (not at all) to 3 (always), with higher scores indicating higher levels of anxiety. The GAS includes three theoretically-derived subscales: Cognitive symptoms, Somatic symptoms, and Affective symptoms. The number of items for each subscale ranges from 8 to 9. The GAS total score is based on the first 25 items. The additional 5 content items assess areas of anxiety often reported to be of concern for older adults (health and financial concerns, fear of dying, and so on). These items are for clinical use alone and therefore do not load on the total GAS score.

The GAS was translated from English into Italian through a 6-stage procedure, including an initial translation and a back-translation process carried out by a group of researchers at the University of Bergamo (Gatti et al., 2017). At stage 1, two bilingual translators with Italian mother tongue carried out an independent forward translation. At stage 2, the two translators and a research group discussed and synthesized the results to develop a single forward translation. At stage 3, two bilingual translators with English mother tongue translated the GAS back into English. At stage 4, all translators (2 forward translators + 2 back translators) together with the research group took part in a focus group discussion. Another expert in geriatric psychology, without any previous knowledge of translation procedures, also participated in the focus group. At stage 5, the pre-final version of the questionnaire was administered to a sample of 15–20 older adults. At stage 6, the research group generated a final report to provide a description of all translations and cultural adaptations made. In the original validation study (Segal et al., 2010), internal consistency of the measure was excellent for the GAS Total score and the 3 Subscales (Total score α = 0.93; Cognitive α = 0.90; Somatic α = 0.80; Affective α = 0.82). Cronbach's alphas for the GAS in the present sample were good: 0.88 for Total score, 0.76 for Cognitive scale, 0.77 for Somatic scale, and 0.75 for Affective scale.

#### Geriatric anxiety inventory (GAI)

The Geriatric Anxiety Inventory (Pachana et al., 2007; Italian version by Rozzini et al., 2009) is a 20-item self-report measure used to assess dimensional anxiety among older adults. It has a dichotomous yes/no response format and therefore provides an easy to use response format for mild cognitively impaired older adults. The total score of the GAI ranges from 0 to 20, with higher scores corresponding to higher levels of anxiety. Its internal consistency has been shown to be excellent in samples of community-dwelling older adults and older adults receiving psychiatric services (Andrew and Dulin, 2007; Pachana et al., 2007; Diefenbach et al., 2009; Byrne et al., 2010). Evidence regarding the concurrent validity of the GAI showed moderate to strong correlations with other anxiety measures (Pachana et al., 2007; Yochim et al., 2011) and worry (Pachana et al., 2007; Diefenbach et al., 2009). Divergent validity with measures of depression varied across studies (*r* = 0.38 in Byrne et al., 2010; *r* = 0.74 in Yochim et al., 2011). The Italian version of the GAI exhibited high test-retest reliability (*r* = 0.86), good internal consistency (Cronbach's alpha = 0.76), as well as a high level of concurrent validity with the Anxiety Status Inventory (ASI, Zung, 1971) (*r* = 0.85) (Rozzini et al., 2009). In the present sample, Cronbach's alpha was 0.90.

#### Teate depression inventory (TDI)

The TDI (Balsamo and Saggino, 2013a; Balsamo et al., 2014b) is a 21-item self-report instrument designed to assess symptoms of Major Depressive Disorder as specified in the latest edition of the DSM (DSM-5, American Psychiatric Association, 2013), in order to overcome psychometric weaknesses of existing measures of depression (Balsamo and Saggino, 2007). It was developed via Rasch logistic analysis of responses, within the framework of Item Response Theory (Rasch, 1960; Andrich, 1995). Each item is rated on a 5-point Likert-type scale, ranging from 0 (always) to 4 (never). Growing literature suggests that the TDI has strong psychometric properties in both clinical and nonclinical samples, including an excellent Person Separation Index, no evidence of bias due to item-trait interaction, good discriminant and convergent validity, and control of major response sets (Balsamo et al., 2013b, 2015a,b,c; Innamorati et al., 2013, 2014; Saggino et al., 2014, 2017; Contardi et al., 2018). Additionally, three cut-off scores were recommended in terms of sensitivity, specificity and classification accuracy for screening for varying levels (minimal, mild, moderate, and severe) of depression severity in a group of patients diagnosed with Major Depressive Disorder (Balsamo and Saggino, 2014a). In the present sample, Cronbach's alpha was 0.88.

#### Big five questionnaire (BFQ-2)

Personality traits were assessed via the Big Five Questionnaire (BFQ-2; Caprara et al., 1993, 2007) which comprises 134 items rated on a 5-point Likert scale (1 = very false for me, 5 = very true for me). The BFQ has been shown to be a valid and reliable measure of the Big Five traits in large samples of Italian respondents as well as in cross-cultural comparisons (e.g., Caprara et al., 2000). In the present study, the internal consistencies of the five traits were 0.83 (for Extraversion), 0.90 (for Agreeableness), 0.83 (for Conscientiousness), 0.91 (for Openness), and 0.89 (for Emotional Stability).

### Data analysis

Factorial structure of the GAS was examined within the framework of structural equation modeling (CFA) analyzed by EQS 6.0 (Bentler, 2006), allowing for correlation among error terms.

The analyses were performed on covariance matrices, since SEM statistical theory relies on the distributional properties of the elements of a covariance matrix.

The method of estimation used in all models was the robust maximum likelihood estimator, which yields corrected standard errors using the Satorra-Bentler method (Satorra and Bentler, 1994; Rhemtulla et al., 2012). Accordingly, we reported the Satorra-Bentler chi square statistic, with the following robust indices: robust comparative fit index (CFI), robust root mean square error of approximation (RMSEA), and robust standardized root-mean-square residual (SRMR). The following heuristic labels were used to describe model fit: acceptable when CFI was 0.90–0.94, RMSEA was 0.08 and SRMR was 0.08, while good when CFI is equal to or above 0.95, RMSEA is 0.06 or below and SRMR is 0.05 (Hu and Bentler, 1998; Yu, 2002; Byrne, 2006; Steiger, 2007). Lagrange multiplier test (LM) was used to identify which fixed parameters, if freely estimated, would lead to a significantly better fitting model. The LM test operates multivariately in determining misspecified parameters in a model. EQS produces univariate and multivariate χ^2^ statistics that permit evaluation of the appropriateness of the specific restrictions; it also yields a parameter change statistic that represents the value that would be obtained if a particular fixed parameter were freely estimated in a future run. Statistically significant LM χ^2^ values would argue for the presence of factor cross-loadings and error covariances, respectively. Decisions regarding possible misspecification followed by respecification of the model are based on the incremental univariate statistics. The user tipically looks for parameters whose χ^2^ values stand apart from the rest and probabilities < 0.05 (Byrne, 2006). We used the Expected Parameter Change (EPC) in combination with the Modification Index (MI) (Saris et al., 2009). For each parameter tested via the LM Test, the parameter change statistic represents its estimated value if this parameter is freely estimated in a subsequent test of the model. If the EPC is rather small, one concludes that there is no serious misspecification. However, when the EPC is large, for example larger than 0.2, it is concluded that there is a relevant misspecification in the model.

In addition, Multigroup Confirmatory Factor Analysis (MG-CFA; Meredith, 1993; van de Schoot et al., 2012) was performed to test measurement invariance of the GAS with respect to gender on a set of nested models, that begin with the separate determination of a baseline model for each group. Estimation is based on the robust statistics (ML, robust; the S-B χ^2^) and analyses are based on the covariance matrix. The intercepts in addition to variances and covariances will be modeled. Associated with each constraint is a cumulative multivariate LM Test χ^2^ value, and an incremental univariate χ^2^ value, along with their probability values. To locate parameters that are noninvariant across groups, we look for probability values associated with the incremental univariate χ^2^ values that are < 0.05. Invariance was tested for configural (M1), metric (M2) and scalar (M3) invariance. According to Cheung and Rensvold (Cheung and Rensvold, 2000), the ΔCFI is a robust statistic for testing the between-group invariance of CFA models. They recommended that invariance can be assumed when this value is 0.01 or less, in absolute values. Finally, the invariance of Latent Factor Means was to be examined in a CFA framework.

We used the value of the critical ratio (CR) to assess latent mean differences. CR is calculated by parameter estimate divided by its standard error, which tests whether the coefficient is significantly different from 0. A CR value larger than 1.96 indicates statistically significant differences in the latent means (Byrne, 2006).

Using IBM SPSS (2010), internal consistency was estimated by Cronbach's alpha (Cronbach, 1951), McDonald's omega (ω; Zinbarg et al., 2005; Dunn et al., 2014), and mean corrected item-total correlations. The homogeneity assumption stating that the population variances are equal for gender was tested by Levene's Test (Barbaranelli, 2006). Corrected item-total correlations were calculated to examine how each item contributed to the overall scale. Cronbach's alpha below 0.60 are unacceptable, whereas item inter-correlation coefficients higher than 0.30 are adequate (Nunnally and Bernstein, 1994).

To assess convergent and discriminant validity, relationships between the GAS total, its subscales, and all other measures were investigated using correlation coefficients (Pearson's *r*). The point biserial correlation (r_pb_) is the value of Pearson's product moment correlation when one of the variables is dichotomous and the other variable is metric. However, when the values of the two categories of the dichotomous variables are 0 and 1, r_pb_ = r (Pearson's) (p. 143, Ercolani et al., 2001). Mathematically, the Point-Biserial Correlation Coefficient is calculated just as the Pearson's Bivariate Correlation Coefficient would be calculated, where in the dichotomous variable of the two variables is either 0 or 1- which is why it is also called the binary variable.

This was followed by application of the Fisher r-to-z transformation (Cohen and Cohen, 1983) to examine one-tailed differences in the magnitude of the correlation coefficients to determine whether correlations were significantly different from each other. If r_a_ is greater than r_b_, the resulting value of z will have a positive sign; if r_a_ is smaller than r_b_, the sign of z will be negative.

## Results

The descriptive statistics of all GAS items, arranged for the three subscales, are presented in Table 2. The means of the 3-point Likert GAS items were relatively low with values ranging from 0.17 (Item 4) to 1.10 (Item 23).

**Table 2:** Descriptive statistics of the Italian GAS (*N* = 346).

	**Item**	**Subscale**	**Mean (*SD*)**	**Min-Max**	**Skewness (*SE*)**	**Kurtosis (*SE*)**
My heart raced or beat strongly.	1	Somatic	0.66 (0.59)	0–2	0.27 (0.13)	−0.66 (0.26)
My breath was short.	2	Somatic	0.63 (0.60)	0–2	0.38 (0.13)	−0.67 (0.26)
I had an upset stomach.	3	Somatic	0.53 (0.61)	0–2	0.71 (0.13)	−0.45 (0.26)
I had difficulty falling asleep.	8	Somatic	0.91 (0.80)	0–3	0.59 (0.13)	−0.15 (0.26)
I had difficulty staying asleep.	9	Somatic	0.77 (0.81)	0–3	0.75 (0.13)	−0.24 (0.26)
I had a hard time sitting still.	17	Somatic	0.33 (0.56)	0–2	1.45 (0.13)	1.15 (0.26)
I felt tired.	21	Somatic	1.08 (0.67)	0–3	0.38 (0.13)	0.45 (0.26)
My muscles were tense.	22	Somatic	0.64 (0.67)	0–3	0.73 (0.13)	0.22 (0.26)
I had back pain, neck pain, or muscle cramps.	23	Somatic	1.10 (0.78)	0–3	0.41 (0.13)	−0.09 (0.26)
I felt like things were not real or like I was outside of myself.	4	Cognitive	0.17 (0.38)	0–1	1.73 (0.13)	0.99 (0.26)
I felt like I was losing control.	5	Cognitive	0.29 (0.48)	0–2	1.23 (0.13)	0.26 (0.26)
I had difficulty concentrating.	12	Cognitive	0.65 (0.56)	0–2	0.14 (0.13)	−0.74 (0.26)
I felt like I was in a daze.	16	Cognitive	0.25 (0.45)	0–2	1.47 (0.13)	0.90 (0.26)
I worried too much.	18	Cognitive	0.82 (0.67)	0–3	0.34 (0.13)	−0.29 (0.26)
I could not control my worry.	19	Cognitive	0.57 (0.73)	0–3	1.24 (0.13)	1.20 (0.26)
I felt like I had no control over my life.	24	Cognitive	0.18 (0.38)	0–1	1.71 (0.13)	0.92 (0.26)
I felt like something terrible was going to happen to me.	25	Cognitive	0.25 (0.47)	0–2	1.63 (0.13)	1.72 (0.26)
I was afraid of being judged by others.	6	Affective	0.53 (0.59)	0–2	0.62 (0.13)	−0.55 (0.26)
I was afraid of being humiliated or embarrassed.	7	Affective	0.32 (0.53)	0–2	1.34 (0.13)	0.84 (0.26)
I was irritable.	10	Affective	0.75 (0.58)	0–2	0.10 (0.13)	−0.46 (0.26)
I had outbursts of anger.	11	Affective	0.48 (0.60)	0–2	0.84 (0.13)	−0.28 (0.26)
I was easily startled or upset.	13	Affective	0.41 (0.56)	0–2	0.99 (0.13)	0.01 (0.26)
I was less interested in doing something I typically enjoy.	14	Affective	0.45 (0.58)	0–2	0.87 (0.13)	−0.24 (0.26)
I felt detached or isolated from others.	15	Affective	0.34 (0.53)	0–2	1.24 (0.13)	0.55 (0.26)
I felt restless, keyed up, or on edge.	20	Affective	0.62 (0.65)	0–2	0.57 (0.13)	−0.65 (0.26)
Somatic subscale	9		6.65 (3.64)	0–18	0.42 (0.13)	−0.13 (0.26)
Cognitive subscale	8		3.17 (2.60)	0–11	0.71 (0.13)	−0.28 (0.26)
Affective subscale	8		3.91 (2.79)	0–12	0.51 (0.13)	−0.48 (0.26)

*Somatic subscale (9 items) = sum of items 1, 2, 3, 8, 9, 17, 21, 22, 23. Cognitive subscale (8 items) = sum of items 4, 5, 12, 16, 18, 19, 24, 25. Affective subscale (8 items) = sum of items 6, 7, 10, 11, 13, 14, 15, 20; SD = Standard Deviation; SE = Standard error*.

Inspection of skewness and kurtosis indexes indicated that departures from normality were not severe, so no variable transformations were deemed necessary (West et al., 1995).

### Confirmatory factor analysis, invariance measurement and invariance of latent factor means

Prior to model testing, Mardia's test of normality was used to assess the normality of data by evaluating the kurtosis (Mardia's normalized estimate = 798.113; Mardia, 1974). The high Mardia's normalized estimate of kurtosis suggested non full normality of data. Thus, all analyses were based on the robust maximum likelihood estimator (Satorra and Bentler, 1994).

Confirmatory factor analysis (CFA) was used to validate both the originally postulated three factor structure of the GAS (Model 1: Cognitive, Affective and Somatic; Segal et al., 2010), a one general anxiety factor solution (Model 2), and to test the two-factor structure (Model 3), found by Picconi, Balsamo and Fairfield (report not published, 2017)^1^, through a Principal Axis Factoring (PAF) with Direct Oblimin rotation, in which Cognitive/Affective and Somatic factors emerge (see Table 3). Goodness-of-fit statistics for all tested structural models were presented in Table 4. The SB χ^2^ goodness-of-fit tests were significant for each of the CFA models (SB χ^2^ ranged from 431.80, *df* = 271, to 406.15, *df* = 269, *p* < 0.001).

**Table 3:** Factor structure extracted–Efa.

**ITEM**	**Extracted factors and loadings (*****n*** = **346)**		***h*^2^**
	**COGNITIVE/AFFECTIVE**	**SOMATIC**	
Item 5. I felt like I was losing control.	**0.664**	−0.072	0.394
Item 24. I felt like I had no control over my life.	**0.612**	−0.080	0.328
Item 25. I felt like something terrible was going to happen to me.	**0.576**	0.007	0.336
Item 7. I was afraid of being humiliated or embarrassed.	**0.557**	−0.049	0.283
Item 6. I was afraid of being judged by others.	**0.549**	−0.076	0.261
Item 15. I felt detached or isolated from others.	**0.531**	−0.046	0.258
Item 13. I was easily startled or upset.	**0.475**	0.162	0.336
Item 19. I could not control my worry.	**0.460**	0.260	0.409
Item 4. I felt like things were not real or like I was outside of myself.	**0.429**	−0.042	0.166
Item 16. I felt like I was in a daze.	**0.421**	0.110	0.239
Item 11. I had outbursts of anger.	**0.400**	0.118	0.225
Item 14. I was less interested in doing something I typically enjoy.	**0.325**	0.082	0.141
Item 12. I had difficulty concentrating.	0.297	0.222	0.209
Item 10. I was irritable.	0.272	0.245	0.206
Item 8. I had difficulty falling asleep.	−0.061	**0.672**	0.411
Item 21. I felt tired.	−0.030	**0.618**	0.363
Item 9. I had difficulty staying asleep.	−0.059	**0.594**	0.318
Item 22. My muscles were tense.	0.048	**0.576**	0.364
Item 23. I had back pain. neck pain. or muscle cramps.	−0.054	**0.516**	0.239
Item 3. I had an upset stomach.	0.033	**0.440**	0.211
Item 20. I felt restless, keyed up, or on edge.	**0.380**	**0.423**	0.497
Item 18. I worried too much.	**0.353**	**0.379**	0.413
Item 2. My breath was short.	0.177	**0.326**	0.200
Item 1. My heart raced or beat strongly.	0.293	**0.322**	0.291
Item 17. I had a hard time sitting still.	0.183	0.232	0.133
% explained variance	0.162	0.127	**0.289**

*h^2^ is communality. All factor loadings of ≥ 0.30 are in bold; % of variance explained is in bold*.

**Table 4:** Fit indices for the structural models (*N* = 346).

**MODEL**	**SB χ2**	***df***	**CFI**	**SRMR**	**RMSEA**		**90% CI**	**AIC**
Three theoretical factor (Model 1)	406.15^*^	269	0.93	0.053	0.038		0.031/0.046	−131.85
One factor (Model 2)	418.84^*^	248	0.91	0.057	0.045		0.037/0.052	−77.15
Two factor, cognitive/affective-somatic (Model 3)	431.80^*^	271	0.91	0.055	0.041		0.034/0.049	−110.19
**FACTOR LOADINGS, STANDARDIZED SOLUTION AND FACTOR STRUCTURE COEFFICIENTS (R**_s_**) -MODEL 1**							
		**Somatic pattern (R**_s_**)**		**Cognitive pattern (R**_s_**)**		**Affective pattern (R**_s_**)**	
Item 1. My heart raced or beat strongly.		0.586 (0.432)		0		0 (0.430)	
Item 2. My breath was short.		0.511 (0.377)		0		0 (0.375)	
Item 3. I had an upset stomach.		0.465 (0.343)		0		0 (0.341)	
Item 8. I had difficulty falling asleep.		0.521 (0.384)		0		0 (0.382)	
Item 9. I had difficulty staying asleep.		0.439 (0.324)		0		0 (0.322)	
Item 17. I had a hard time sitting still.		0.375 (0.277)		0		0 (0.275)	
Item 21. I felt tired.		0.583 (0.430)		0		0 (0.427)	
Item 22. My muscles were tense.		0.626 (0.462)		0		0 (0.459)	
Item 23. I had back pain, neck pain, or muscle cramps.		0.502 (0.375)		0		0 (0.368)	
Item 4. I felt like things were not real or like I was outside of myself.		0		0.358 (0.264)		0 (0.343)	
Item 5. I felt like I was losing control.		0		0.560 (0.413)		0 (0.536)	
Item 12. I had difficulty concentrating.		0		0.440 (0.325)		0 (0.421)	
Item 16. I felt like I was in a daze.		0		0.480 (0.354)		0 (0.459)	
Item 18. I worried too much.		0		0.689 (0.508)		0 (0.659)	
Item 19. I could not control my worry.		0		0.688 (0.508)		0 (0.658)	
Item 24. I felt like I had no control over my life.		0		0.458 (0.338)		0 (0.438)	
Item 25. I felt like something terrible was going to happen to me.		0		0.530 (0.391)		0 (0.507)	
Item 6. I was afraid of being judged by others.		0 (0.309)		0 (0.404)		0.422	
Item 7. I was afraid of being humiliated or embarrassed.		0 (0.328)		0 (0.428)		0.447	
Item 10. I was irritable.		0 (0.356)		0 (0.464)		0.485	
Item 11. I had outbursts of anger.		0 (0.380)		0 (0.496)		0.518	
Item 13. I was easily startled or upset.		0 (0.430)		0 (0.560)		0.586	
Item 14. I was less interested in doing something I typically enjoy.		0 (0.270)		0 (0.352)		0.368	
Item 15. I felt detached or isolated from others.		0 (0.332)		0 (0.433)		0.453	
Item 20. I felt restless, keyed up, or on edge.		0 (0.549)		0 (0.717)		0.749	

* *p < 0.001*.

*SB χ^2^, Satorra and Bentler chi-squared test; df, degrees of freedom; CFI, comparative fit index; SRMR, standardized root mean square residual; RMSEA, root-mean-square error of approximation; 90% CI, 90% confidence interval of RMSEA; AIC, Akaike's information criterion used in the comparison of two or more models with smaller values representing a better fit of the hypothesized model (Hu and Bentler, 1995); Pattern coefficients constrained and not estimated in the model are presented as “0”; the structure coefficients are added in parentheses next to the pattern coefficients*.

Together, results supported both the two factor Cognitive/Affective and Somatic and the one factor solution implied by the GAS item pool.

However, Model 1 (three factor structure) demonstrated significantly better fit compared to Model 2 (one general anxiety factor solution) (Satorra-Bentler Scaled Chi-Square Difference = 6.84; *df* = 21; *p* = 0.998) (Brown, 2006; Satorra and Bentler, 2010; Barbaranelli and Ingoglia, 2013), and respect to Model 3 (two-factor structure) (Satorra-Bentler Scaled Chi-Square Difference = 48.14; *df* = 2; *p* < 0.001), with the presence of three error covariances between the items (GAS9 and GAS8, GAS7 and GAS6, GAS25 and GAS24), suggested by Lagrange multiplier test (MI) and by the expected parameter change statistic (EPC) (Saris et al., 1987). Factor loadings, standardized solution of the items and factor structure coefficients, which can be essential for the accurate interpretation of CFA results, are shown in Table 4 (Graham et al., 2003).

In Model 1, all factor loadings were statistically significant and ranged from 0.36 to 0.75, with an average standardized factor loading of 0.51. Squared multiple correlations ranged from 0.13 to 0.56, with an average SMC of 0.27 indicating that, on average, 27% of the variance in observed variables was accounted for by latent factors. The latent factor correlations were very high, ranging between 0.73 and 0.96. We added also the structure coefficients, which are merely the correlations between the measured variables and the latent factors. Measured variables are correlated with *all* factors when the factors are correlated, even for variables with CFA pattern parameters fixed to be zeroes. The estimation of these structure coefficients does not cost additional degrees of freedom, since the coefficients are fully determined by the pattern and the factor correlation coefficients already being estimated. The structure coefficients are analogous to the zero-order bivariate Pearson correlations without isolating the overlapping relationships among the factors (Thompson, 1997; Graham et al., 2003).

Then, a multiple-group approach was used to test measurement invariance across gender (see Table 5).

**Table 5:** Test for measurement invariance of the GAS across gender: Summary of goodness of fit statistics.

**Model**	**SB χ2**	**df**	**CFI**	**RMSEA**	**RMSEA 90% CI**	**Model Comparison**	**Δ CFI**
Baseline model males	356.4657	268	0.913	0.044	0.031, 0.055	- - - - - - - - - -	- - - - - - -
Baseline model females	360.0953	270	0.903	0.044	0.031, 0.055	- - - - - - - - - -	- - - - - - -
M1	716.5637	538	0.899	0.031	0.025, 0.037	- - - - - - - - - -	- - - - - - -
M2^*^	740.1375	562	0.899	0.030	0.024, 0.036	2 vs. 1	0
M3	784.9105	587	0.892	0.031	0.025, 0.037	3 vs. 2	0.007

*M1, model for configural invariance; no constraints; M2, model for full metric invariance with all factor loadings constrained equal. M3, model for scalar invariance; with all intercepts constrained equal*.

* *We included the correlation between errors. Equality constrains are specified for two common error covariance GAS9 and GAS8; GAS7 and GAS6, except the two involving GAS2 and GAS1 and GAS25 and GAS24; unique for males*.

Measurement invariance across gender groups was entirely supported at the factorial structure, and at the intercept level. The ΔCFIs are lower than 0.01 in all models, suggesting that invariance can be assumed. Based on the establishment of the full scalar invariance across gender, we can compare the latent mean differences across this group. To obtain an estimate of this difference, the female group was chosen as a reference group. Thus, since the female group was designated as the reference group, their factor means were fixed to zero, and we concentrated solely on estimates as they relate to the male group. Because analyses were based on the robust statistics, these estimates are interpreted in terms of robust standard errors and the resulting z-statistics. Accordingly, these results indicate that whereas the means of F1 (Somatic; females = 7.13; males = 6.17; CR = −2.246; small effect size, Cohen's d^2^ = −0.27) and F3 (Affective; females = 4.20; males = 3.62; CR = −2.128; small effect size, Cohen's *d* = −0.21) for males were significantly different from those for females, the means for F2 (Cognitive; females = 3.35; males = 3.00; *CR* = −1.332; zero o near zero effect, Cohen's *d* = −0.14) were not. More specifically, considering the negative signs associated with these statistically significant values, the finding showed that F1 and F3 for males appeared to be less positive on average than for females.

A positive CR implies that the comparison group has higher latent mean than the reference group. Conversely, a negative CR suggests that the comparison group's latent mean is smaller than the reference group (Byrne, 2006). The population variances are equal for all gender groups (*p* = not significant).

### Reliability

Internal consistency of the subscales was good: α = 0.76 (95% Confidence Interval: Lower Bound = 0.722; Upper Bound = 0.798; *p* < 0.001; ω = 0.81; Cognitive), α = 0.77, (95% Confidence Interval: Lower Bound = 0.732; Upper Bound = 0.805; *p* < 0.001; ω = 0.82; Somatic) and α = 0.75 (95% Confidence Interval: Lower Bound = 0.705; Upper Bound = 0.786; *p* < 0.001; ω = 0.83; Affective). Analysis using Feldt's test (see Feldt, 1969; Feldt et al., 1987) indicating that the Cronbach's alpha doesn't significantly differ.

According to the corrected item-total correlations, no items appeared less suitable as indicators of their respective construct. This means that no item correlations with the scale, excluding the item itself, fall in the low range of 0.0-0.3, and discriminated well (Kline, 1986; Barbaranelli and Natali, 2005; Barbaranelli and D'Olimpio, 2007). The inter-correlations mean of items within each scale ranged from 0.47 (Cognitive) to 0.44 (Affective).

### Scale intercorrelations

As expected, the GAS total scale was positively and strongly correlated with the Cognitive subscale (see Table 6; *r* = 0.86, *p* < 0.001, 74% variance shared), Affective subscale (*r* = 0.85, *p* < 0.001, 72% variance shared), and Somatic subscale (*r* = 0.85, *p* < 0.001, 72% variance shared). As expected, the three subscales were highly correlated, with r varying from 0.52 to 0.71 (*p* < 0.001). In addition, the correlation between the Cognitive and Affective subscales was stronger (*r* = 0.71, *p* < 0.001, 50% variance shared) than the correlation between the Cognitive and Somatic subscale (*r* = 0.56, *p* < 0.001, 31% variance shared) and between the Affective and Somatic subscale (*r* = 0.52, *p* < 0.001, 27% variance shared).

**Table 6:** GAS inter-scale correlations (*n* = 346), correlations with convergent (GAI, *n* = 346; Emotional Stability, *n* = 345) and discriminant scales (TDI, *n* = 327; Extraversion, Openness, Agreeableness, Conscientiousness, *n* = 345).

	**S**	**C**	**A**
Somatic (S)	1		
Cognitive (C)	0.56^***^	1	
Affective (A)	0.52^***^	0.71***	1
GAI	0.82^***^	0.85^***^	0.83^***^
TDI	0.39^***^	0.45^***^	0.41^***^
Extraversion	−0.09	−0.17^***^	−0.01
Openness	−0.17^***^	−0.17^***^	−0.10
Agreeableness	0.03	−0.07	−0.10
Conscientiousness	−0.16x^***^	−0.19^***^	−0.14^*^
Emotional Stability	−0.33^***^	−0.42^***^	−0.48^***^

*S, Somatic Subscale; C, Cognitive Subscale; A, Affective Subscale; GAI, Geriatric Anxiety Inventory; TDI, Teate Depression Inventory*.

* *p < 0.05*;

*** *p < 0.001*.

### Convergent and discriminant validity of the gas

To investigate the convergent and discriminant validity of the Italian version of the GAS, correlations among the GAS total and its subscales with measures of depression, anxiety and personality were computed (see Table 6).

The correlation of the depression scale (TDI) with all anxiety dimensions was weaker than the correlation between measure of anxiety (TDI with GAI, *r* = 0.48). As seen in Table 5, the GAS total score and GAS subscale scores were significantly positively correlated with the TDI, with medium effect sizes (GAS total, *r* = 0.49; Cognitive, *r* = 0.45; Somatic, *r* = 0.39; Affective, *r* = 0.41) and with the GAI with high effect size (GAS total, *r* = 0.97; Cognitive, *r* = 0.85; Somatic, *r* = 0.82; Affective, *r* = 0.83).

Compared to the anxiety scale (GAI), the correlation of 0.49 between the GAS total and the TDI was significantly lower than the correlation of 0.97 between the GAS total and GAI (*z* = −21.04, *p* < 0.001). The correlation of 0.39 between the Somatic subscale and the TDI was significantly lower than the correlation of 0.82 between the Somatic subscale and the GAI, (*z* = −9.48, *p* < 0.001). The correlation of 0.45 between the Cognitive subscale and TDI was significantly lower than the correlation of 0.85 between the Cognitive subscale and the GAI, (*z* = −9.96, *p* < 0.001). The correlation of 0.41 between the Affective subscale and the TDI was significantly lower than the correlation of 0.83 between the Affective and GAI, (*z* = −9.53, *p* < 0.001). Also, GAS total score and GAS subscale scores were substantially correlated with Emotional Stability (GAS total, *r* = −0.47; Cognitive, *r* = −0.42; Somatic, *r* = −0.33; Affective, *r* = −0.48).

However, the discriminant correlations with the other subscales of the BFQ-2 were rather low and only a few appeared to be significant (*p* < 0.001) (GAS total, ranging from *r* = −0.19 for Conscientiousness to *r* = −0.04 for Agreeableness; Cognitive, ranging from *r* = −0.19 for Conscientiousness to *r* = −0.07 for Agreeableness; Somatic, ranging from *r* = −0.16 for Conscientiousness to *r* = 0.03 for Agreeableness; Affective, ranging from *r* = −0.14 for Conscientiousness to *r* = −0.01 for Extraversion).

### Gas content items

Finally, as stated above, the GAS includes five additional content items (items 26-30) that do not load on any scales but are used for clinical purposes and provide information about areas of anxiety often reported to be of concern for older adults (e.g., fear of dying, financial or health concerns; Segal et al., 2010). These scores are not included in the GAS total score.

A rank order of the means of these five content items showed that item 28 was the highest ranked item (“*I was concerned about my children*”, *M* = 1.50, *SD* = 1.02), followed by item 27 (“*I was concerned about my health”, M* = 1.01, *SD* = 0.75), item 26 (“*I was concerned about my finances”, M* = 0.80, *SD* = 0.79), item 30 (“*I was afraid of becoming a burden to my family or children*”, *M* = 0.63, *SD* = 0.83) and item 29 (“*I was afraid of dying*”, *M* = 0.35, *SD* = 0.55).

## Discussion

This study aimed to examine the psychometric properties of the GAS, translated into Italian, among a larger, geographically more varied sample of older adults. Factor structure, internal reliability, convergent and discriminant validity as well as the gender differences were examined.

Regarding the analysis of the GAS factor structure, the CFA confirmed the better fit of the three factors (Cognitive, Somatic, and Affective) originally derived from English version (Segal et al., 2010; Yochim et al., 2011, 2013). The three latent factors are those that best explained the data.

The GAS captured the broad range of anxiety disorder symptoms. The clinician or researcher can easily determine which types of symptoms are more problematic for the respondent (Segal et al., 2010).

Results also provided evidence about gender invariance. The test of the metric and scalar invariance of the model in relation to gender revealed that all the factor loadings showed to be invariant and the intercepts for observed variables loading on the same latent variable. As scalar invariance was established, means can be reliably compared. Sex is a variable which has been identified as a risk factor for anxiety. Analyses of latent mean differences revealed that females exhibited higher means than males on two GAS subscales, Somatic and Affective, where the means for Cognitive Factor were not.

As expected, women tend to report higher levels of anxiety than men, a finding that is reported consistently in literature. Gum et al. (2009) found that community-dwelling individuals who were diagnosed with an anxiety disorder were more likely to be female. Furthermore, female gender has been associated with a greater likelihood of anxiety chronicity in older adults (De Beurs et al., 2000; Gatti et al., 2017), such that anxiety tends to persist in older women compared to older men.

In addition, results suggested that the GAS total score and subscale scores have good internal consistency reliability (Cronbach's alpha, McDonald's omega, and inter-item correlations mean of items). The Cronbach's alpha values compared to values of the Segal et al. (2010) original version did not differ significantly except for the GAS total score (Feldt test = 0.5833, *p* < 0.001; see Feldt, 1969; Feldt et al., 1987) and Cognitive scale (Feldt test = 0.4167, *p* < 0.001) in which the original sample scored higher reliabilities values. Similar results were found when comparing the alpha values of the Italian version of the GAS with both the Persian and German versions. Cronbach's alpha values not differ significantly (all *p* = ns; Feldt et al., 1987).

Regarding interscale correlations, as expected, there were strong positive relationships between the GAS total score and each of the GAS subscales. Therefore, the relatively high intercorrelation of the scales, which especially occurred between the Cognitive and Affective subscales, is not surprising and can be traced back to the fact that symptoms of anxiety disorders are often comorbid with each other (DSM-IV-TR and DSM-V; Kogan et al., 2000; Segal et al., 2010; Wolitzky-Taylor et al., 2010; American Psychiatric Association, 2013).

Convergent validity of the GAS was evidenced via significant and high correlations between the GAS total score, subscale scores and another measure of anxiety (GAI).

With respect to the discriminant validity of the GAS, our findings confirmed the expected low relationships with measures of constructs that are non-related (i.e., Extraversion, Openness, Agreeableness, Conscientiousness), or negatively related (i.e., Emotional Stability) to anxiety, whereby the relation between the GAS total score, subscale scores and depression (TDI) was lower than the correlation with anxiety measure. Anxiety in older adults is highly co-morbid with depressive symptoms (Beekman et al., 2000).

It is not surprising that Cognitive subscale and following Affective subscale were associated with measure of depression more strongly than Somatic subscale, because cognitive and affective aspect are two important components of many anxiety disorders (Cioffi et al., 2008; Van Dam et al., 2013; Balsamo et al., 2015b).

Together, the present findings support the reliability and validity of the GAS as a measure of anxiety in an Italian geriatric population. These results are important because the detection of anxiety in older adults is generally complicated by the high frequency of medical disorders present in this age group (Balsamo et al., 2015b, 2018).

Several limitations of this study should be addressed in future studies.

First, we did not investigate various aspects of reliability of the questionnaire (e.g., test-retest reliability). Second, our results are based on a general community sample of older adults, which limit the generalizability of these findings to clinical conditions. More specifically, the confirmatory models and the correlational analyses among self-report measures found in nonclinical samples might not be similar to the processes in clinical samples (see, for example, Balsamo, 2013; Balsamo et al., 2013c). In addition, our sample is non-representative of the Italian population. More heterogeneous individuals by age, education level and geographical provenience education level and geographic origin would reduce potential selection biases our data could be affected. Therefore, validity and usefulness of the GAS in clinical samples and non clinical are not fully guaranteed. Lastly, correlations for convergent and discriminant validity could be computed by using the SEM approach in order to control over the measurement error, obtaining higher precision than the computation with Pearson's *r*, and other concurrent measures should be taken into consideration, such as measures of trait and state anxiety (Balsamo et al., 2016).

Further research should explore the psychometric performance (e.g., its Differential Item Functioning analysis) of the Italian GAS in larger and more diverse samples of Italian older adults, including also clinical samples and groups with more diverse ethnicity, in order to improve the knowledge on this instrument, providing a more specific assessment of cognitive, affective and somatic anxiety symptoms among older adults. Moreover, a hierarchical or bifactor factorial model could be applied to empirically verify the general score of the GAS in future studies (Reis et al., 2007).

In addition, further studies could be conducted to create a short form of the measure such as Mueller et al. (2015). Short forms of screening measures are preferable in busy clinical settings and in lengthy research protocols to reduce the burden of administration time and scoring.

Because the GAS is based on DSM symptoms of anxiety, it can help clinicians arrive at an accurate diagnosis of an anxiety disorder and thus aid in clinically appropriate treatment.

## Author contributions

All authors listed have made a substantial, direct and intellectual contribution to the work, and approved it for publication.

### Conflict of interest statement

The authors declare that the research was conducted in the absence of any commercial or financial relationships that could be construed as a potential conflict of interest.

## Footnotes

^1^All technical data is available from the authors.

^2^Effect sizes were estimated by Cohen's *d*, where 0.2 is indicative of a small effect, 0.5 a medium and 0.8 a large effect size (Cohen, 1992).
